# Post-Covid mucormycosis presenting as retropharyngeal abscess: a rare case report

**DOI:** 10.1186/s43163-022-00274-2

**Published:** 2022-07-15

**Authors:** Rajat Jain, Devisha Agarwal, Abhishek Bahadur Singh, Veerendra Verma, Hitendra Prakash Singh, Sunil Kumar

**Affiliations:** 1 ENT Department, King George Medical University, Lucknow, India; 2 Department of Otorhinolaryngology and Head Neck Surgery, King George Medical University, Lucknow, Uttar Pradesh India

**Keywords:** Retropharyngeal, Mucormycosis, Covid era, Case report

## Abstract

**Background:**

Post-Covid retropharyngeal mucormycosis is a rare presentation, and no case has been reported in literature until date.

**Case presentation:**

A-32-year-old female post Covid presented to our OPD with history of dysphagia and with a history of steroid intake. Radiology confirmed it as retropharyngeal abscess. Endoscopic-guided aspiration was done. HPE (histopathological examination) revealed classic broad aseptate hyphae of mucormycosis. Patient was managed conservatively with broad-spectrum antifungal.

**Conclusion:**

Retropharyngeal mucormycosis is a rare entity in Covid era. Rapid diagnosis and management are needed to save life of an individual, or results could be fatal.

## Background

Retropharyngeal abscess is a rare entity. It can also be potentially life-threatening. It is most commonly seen in children under the age of five but also seen in adults. Children less than 5 years of age have high propensity towards URTI (upper respiratory tract infection) which may lead to suppurative cervical lymphadenitis and eventually retropharyngeal abscess. In adults, trauma to posterior pharynx may result in retropharyngeal infection leading to abscess formation. Tubercular infection is the main cause of chronic retropharyngeal abscess. Treatment ranges from prolonged courses of intravenous antibiotics to surgical incision and drainage. Mucormycosis was described by Paltauf in 1885 [1]. It is a fungal infection mainly affecting immunocompromised patients and diabetics. Common varieties of mucormycosis are those involving the paranasal sinuses (39%), lungs (24%), skin (19%), brain (9%) and gastrointestinal system (7%) [2]. Cutaneous variety of mucormycosis is a rare entity. Once left untreated, it may become life-threatening due to involvement of critical structures. Mucormycosis is caused by a fungus of the order Mucorales. It is usually harmless to a healthy person but fatal in immunocompromised. Most common causative organism is *Rhizopus* species [3]. Diagnosis is made by histopathological examination, and it is treated by aggressive surgical debridement and appropriate systemic antifungal treatment. It is very rarely found in the retropharyngeal region, and such a case has not yet been reported in literature. We are reporting a case of post-Covid mucormycosis presenting as retropharyngeal abscess — a rare entity.

## Case presentation

**Fig. 1:**
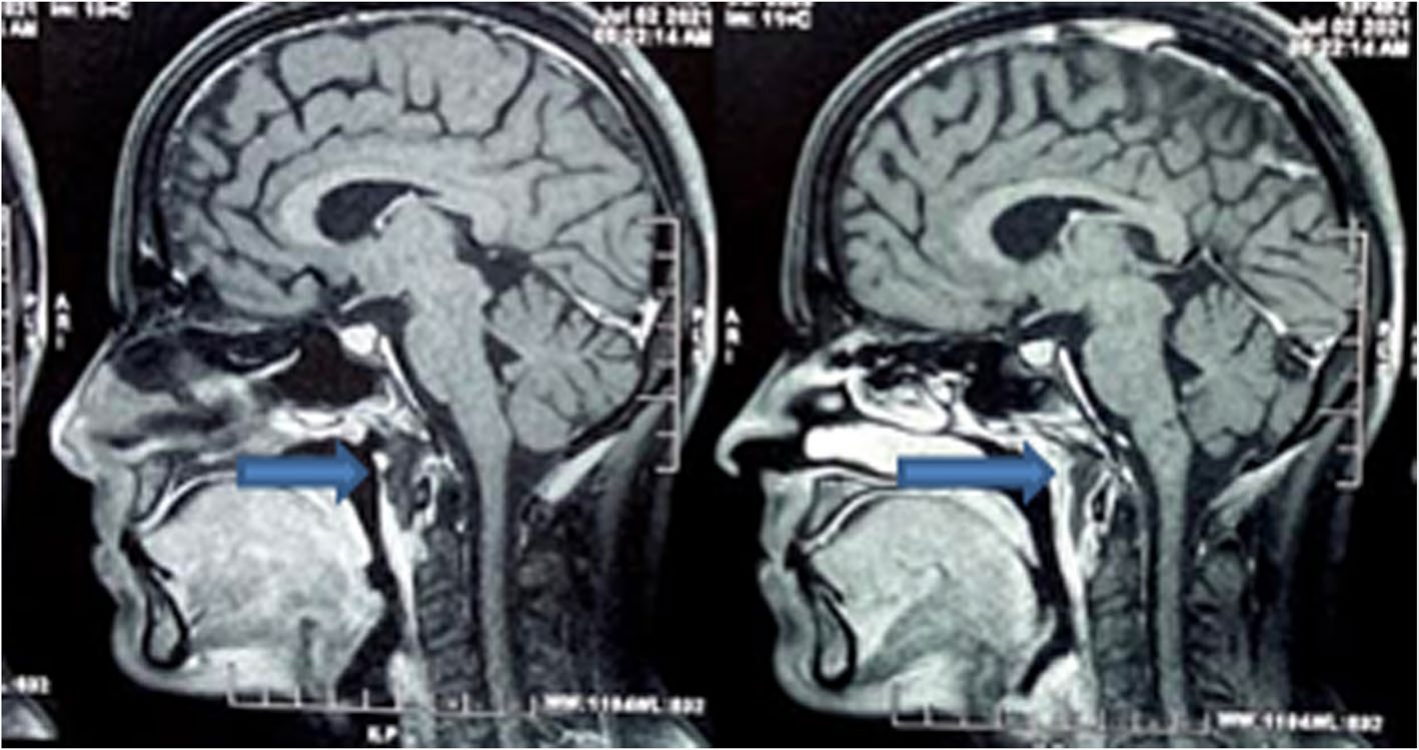
T1 contrast MRI image (Sagittal section) showing retropharyngeal abscess with no contrast uptake

**Fig. 2:**
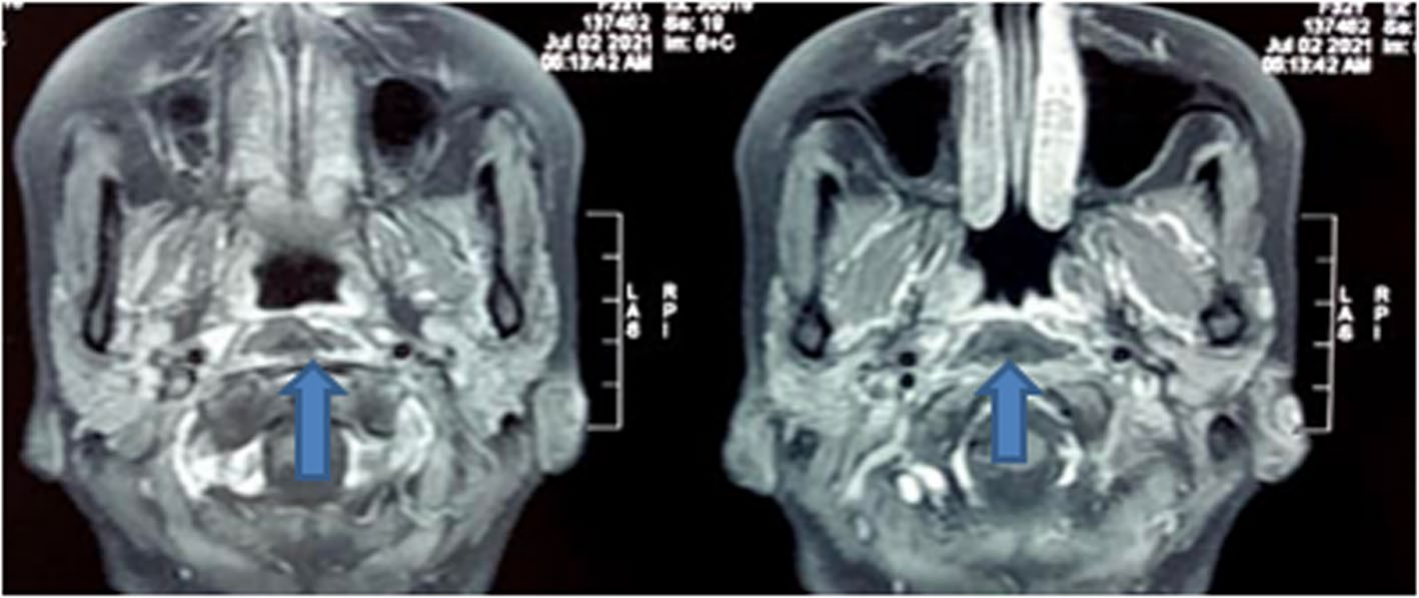
T1 contrast MRI image (Axial section) showing retropharyngeal abscess with no contrast uptake

A 32-year-old female with history of steroid intake following Covid infection presented in the emergency department with dysphagia and odynophagia for the last 4 days. On examination, there was a mild swelling over the face with a minimal retropharyngeal bulge. Diagnostic nasal endoscopy and indirect laryngoscopy were unequivocal. She was a known diabetic with poor control and having HbA1c of 9.4. The WBC counts and serum creatinine levels were also elevated indicating an infective pathology. MRI imaging showed retropharyngeal abscess with no contrast uptake on T1 sequence (axial and sagittal) (Figs. 1, 2).

**Fig. 3:**
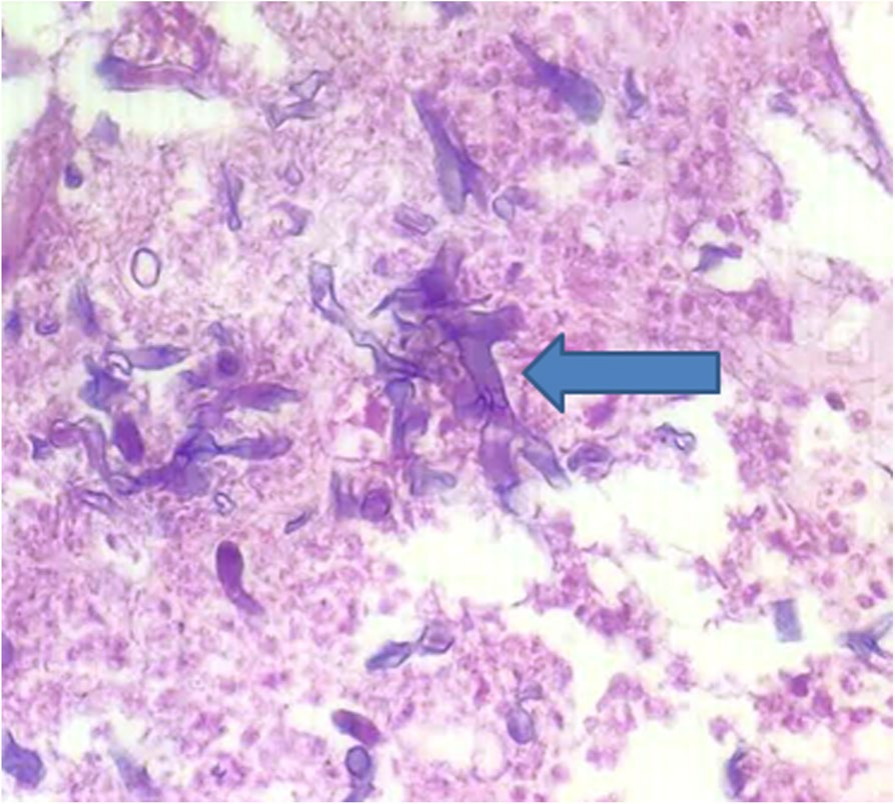
HPE image showing broad, aseptate and obtuse angle branched fungal hyphae—suggestive of mucormycosis

**Fig. 4:**
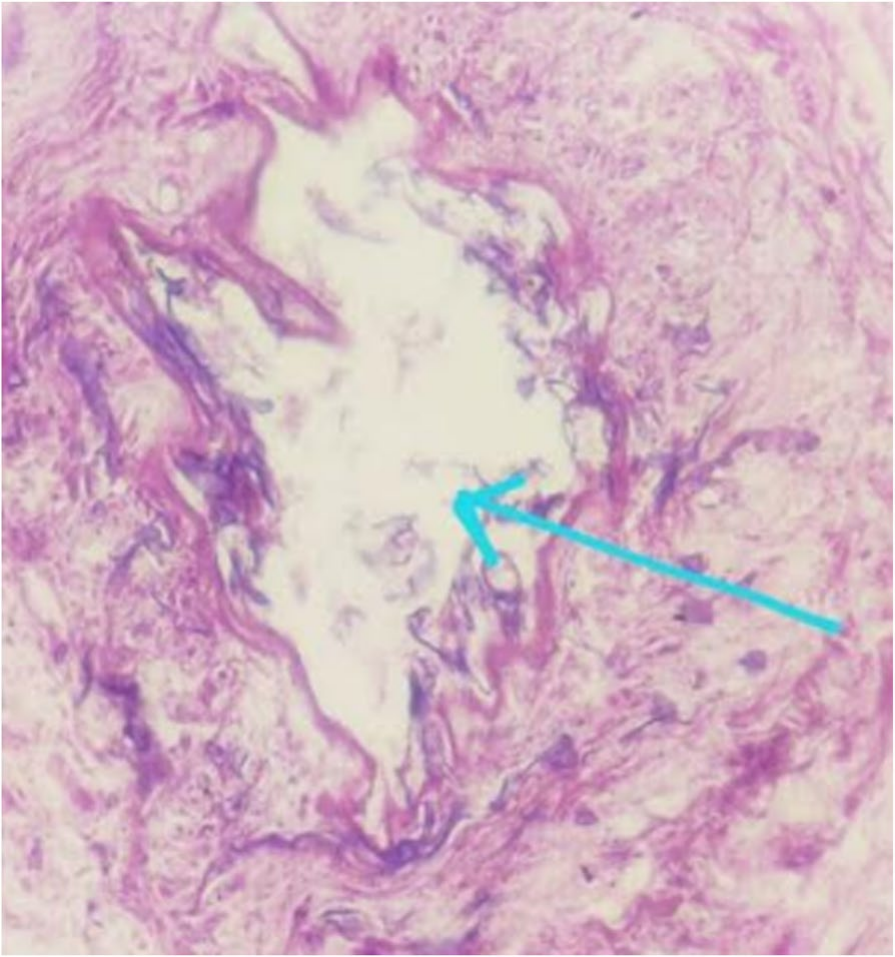
HPE image showing broad, aseptate and obtuse angle branched fungal hyphae with angioinvasion -suggestive of mucormycosis

Endoscopic-guided aspiration of the abscess was done. Pus was sent for gene expert, KOH mount and HPE (histopathological examination). Gene expert denied the presence of *Mycobacterium* tuberculosis. HPE report showed broad, aseptate and obtuse angle branched fungal hyphae—suggestive of mucormycosis (Fig. 3) with angioinvasion (Fig. 4). The sputum for AFB stain was negative.

A multidisciplinary team meeting was conducted with endocrinology and infection control departments. Consensus was reached to treat the patient conservatively with antifungal agents supplemented by broad-spectrum antibiotics. Ryles tube was placed for maintaining nutrition as the patient had difficulty in swallowing. The patient was also advised regular mouth gargles using hydrogen peroxide in 1:4 dilution along with povidone iodine 0.5% w/v 3–4 times a day.

Once the diagnosis of invasive mucormycosis involving retropharyngeal space was made, parenteral liposomal amphotericin B — 5 mg/kg/day along with broad-spectrum antibiotics, was started along with regular monitoring of serum electrolytes and kidney function tests [4]. A total dose of 5 g of liposomal amphotericin was given to the patient. Subsequently, the patient was weaned off the Ryle’s tube after 7 days of treatment and was allowed oral feeds. After a complete regimen of 5 g of liposomal amphotericin, the patient was discharged on 300 mg/day posaconazole for a period of 3 months with regular monitoring of liver function tests. After 1 month of the therapy, the patient was completely relieved of all the symptoms and was comfortably taking oral feeds. Patient was followed up with postoperative MRI. Controlling sugar, ferritin levels are the most important factor in prevention of mucormycosis in patient of Covid-19.

## Discussion

Retropharyngeal space is posterior to the pharynx and oesophagus. Its extent is from the base of the skull to the thoracocervical junction. The main component of the retropharyngeal space is areolar fat. There are two spaces between the visceral (buccopharyngeal) and prevertebral fascia which are subdivided by a thin membrane known as the alar fascia. The anterior space is the “true” retropharyngeal space, described in this article, while the posterior one is called danger space. If there is any infection in the danger space, it can lead to mediastinitis; hence, it is called “danger space”. As the retropharyngeal abscess progressively increases in size, it results in gradual upper airway obstruction. If left unattended and untreated, it can eventually lead to asphyxiation. Mortality in these patients can also occur from sepsis [5]. Mucormycosis is a life-threatening invasive fungal infection. It primarily targets immunocompromised individuals with an impaired neutrophilic response. Factors increasing susceptibility are uncontrolled diabetes mellitus, acquired immunodeficiency syndrome, iatrogenic immunosuppression and haematological malignancies, and also those patients who have undergone organ transplantation [6]. The presence of hyphal invasion of sinus tissue is a characteristic feature of mucormycosis [7, 8]. Clinical presentation of rhinocerebral mucormycosis is similar to complicated sinusitis. Atypical features like nasal blockade, crusting, proptosis, facial pain and oedema, ptosis, chemosis and even ophthalmoplegia, with headache and fever, may present. If there is intracranial extension of the disease, various neurological signs and symptoms like headache and vomiting can manifest [9, 10]. A common presentation is a black eschar which is often seen in the nasal cavity or over the hard palate region [11, 12]. The histopathological picture may present as mycotic infiltration of blood vessels. There may be vasculitis with thrombosis, tissue infarction, haemorrhage and acute neutrophilic infiltration [13, 14]. Intra-orbital and intracranial complications up to 50–80 per cent cases are noticed with improper treatment [15]. Even after aggressive medical and surgical intervention, there may be intracranial extension and ultimately mortality [16]. Surprisingly, there has been a sudden surge of mucormycosis patients in Covid era.

Interplay of multiple factors including diabetes mellitus, respiratory illness, immunosuppressive therapy, hospital-acquired infection and systemic immune alterations by Covid-19 infection may cause secondary infections [17, 18]. Covid-19 patients have reported an overexpression of inflammatory cytokines. They have an impaired cell-mediated immunity with decreased cluster of differentiation (CD) 4- and 8-positive T-helper (CD4+ T and CD8+ T) cell counts. This impairment in immunity favours susceptibility to fungal coinfections [19–21]. Excessive steroid usage in the management of Covid-19 also suppresses immunity, thus favouring opportunistic fungal infections to colonize [22].

Complications associated with retropharyngeal abscess include airway obstruction, bronchial erosion, mediastinitis, sepsis, acute respiratory distress syndrome, cranial nerve palsies, oesophageal perforation, erosion into the carotid artery or jugular vein and meningoencephalitis, so it is mandatory to intervene as quickly as possible in terms of medical (antifungal with broad-spectrum antibiotics) and surgical therapy (incision and drainage, debridement).

## Conclusion

Retropharyngeal abscess in association with post-Covid mucormycosis is a rare and life-threatening condition. Uncontrolled diabetes and abrupt use of steroids are two of the main factors aggravating the illness, and therefore, strict monitoring of sugar levels should be done. Once infected, early surgical intervention and intravenous antifungal treatment along with broad-spectrum antibiotics are the keystones of management.

## Data Availability

The datasets used and/or analysed during the current study are available from the corresponding author on reasonable request.
